# Mutant p53 and Cellular Stress Pathways: A Criminal Alliance That Promotes Cancer Progression

**DOI:** 10.3390/cancers11050614

**Published:** 2019-05-02

**Authors:** Gabriella D’Orazi, Mara Cirone

**Affiliations:** 1 Department of Medical Sciences, University ‘G. d’Annunzio’, 66013 Chieti, Italy; 2 Department of Research, IRCCS Regina Elena National Cancer Institute, 00144 Rome, Italy; 3 Department of Experimental Medicine, “Sapienza” University of Rome, 00185 Rome, Italy; 4 Laboratory Affiliated to Pasteur Institute, Italy-Foundation Cenci Bolognetti, 00161 Rome, Italy

**Keywords:** mutant p53 (mutp53), gain-of-function (GOF), autophagy, endoplasmic reticulum (ER) stress, unfolded protein response (UPR), antioxidant response, heat shock protein (HSP), nuclear factor erythroid 2-related factor 2 (NRF2), hypoxia-inducible factor 1 (HIF-1), anticancer therapy

## Abstract

The capability of cancer cells to manage stress induced by hypoxia, nutrient shortage, acidosis, redox imbalance, loss of calcium homeostasis and exposure to drugs is a key factor to ensure cancer survival and chemoresistance. Among the protective mechanisms utilized by cancer cells to cope with stress a pivotal role is played by the activation of heat shock proteins (HSP) response, anti-oxidant response induced by nuclear factor erythroid 2-related factor 2 (NRF2), the hypoxia-inducible factor-1 (HIF-1), the unfolded protein response (UPR) and autophagy, cellular processes strictly interconnected. However, depending on the type, intensity or duration of cellular stress, the balance between pro-survival and pro-death pathways may change, and cell survival may be shifted into cell death. Mutations of p53 (mutp53), occurring in more than 50% of human cancers, may confer oncogenic gain-of-function (GOF) to the protein, mainly due to its stabilization and interaction with the above reported cellular pathways that help cancer cells to adapt to stress. This review will focus on the interplay of mutp53 with HSPs, NRF2, UPR, and autophagy and discuss how the manipulation of these interconnected processes may tip the balance towards cell death or survival, particularly in response to therapies.

## 1. Introduction

An important protagonist in cellular network preventing human cancer development by protecting the cells from DNA damage and oncogenic signals is p53 tumor suppressor, a DNA sequence-specific transcription factor. Following stress stimuli, such as hypoxia or DNA damage, p53 accumulates in the nucleus and becomes activated inducing target genes involved in several different cellular responses, such as growth arrest, senescence, apoptosis, and DNA repair. These activities prevent the establishment of mutations in the future generation of cells, highlighting the role of oncosuppressor p53 as “guardian of the genome” [1,2]. For these reasons, cancer cells try to get rid of a functional wild-type (wt) p53 pathway that is indeed incompatible with neoplastic growth. An intact wtp53 pathway is also functionally at the base of the tumor cell response to anticancer therapies [3]. Thus, wtp53 loss of function may lead to the development of chemoresistance. This is an important factor in anticancer therapeutic failure [4] that urgently needs to be solved to improve the clinical outcome of anticancer treatments. Mechanistically, a variety of different cellular systems contribute to chemoresistance including tumor heterogeneity, drug-inactivation, evasion of apoptosis, enhanced DNA repair and increased drug efflux [5]. *TP53* is the most frequently inactivated tumor suppressor gene in tumors, being mutated in over 50% of human cancer types and indirectly inactivated in many others [6,7]. Majority of p53 mutations are missense mutations (i.e., R175H, R248Q, R273H, R280K) (hereafter referred to as “mutp53”), classified as structural/conformational and DNA-contact mutations that lead to the synthesis of p53 proteins unable to bind the target gene promoters of wtp53 [8]. In addition, mutp53 can sequester various tumor suppressors including p53 itself (dominant-negative function) and the family members p63 and p73 inhibiting their pro-apoptotic function [9]. Mouse models of different hotspot mutp53 and clinical data from germline and sporadic cancers have clearly established that p53 missense mutations not only abolish the tumor suppressive function but may also acquire new tumorigenic driver activities, namely, gain-of-function (GOF) [10,11]. The best described mechanism of mutp53 GOF is its ability to interact with other transcription factors including NF-Y, Sp1, ETS1/2, NF-kB and SMADs [12], and this interaction profoundly changes the cancer cell transcriptome and proteome, supporting cancer cell survival, tumor progression, invasion, metastasis and chemoresistance [13,14,15]. As a result, cancer cells develop an addiction to these mutp53 oncogenic functions to survive and proliferate. Given their proliferative nature due to oncogene activation, cancer cells undergo various forms of intrinsic stress and adverse environmental challenges, such as oxidative, electrophilic, proteotoxic, inflammatory stress, and nutrient deprivation, that try to manage by activating molecular/cellular pathways, such as autophagy, heat shock protein (HSP), antioxidant response by nuclear factor erythroid 2-related factor 2 (NRF2), endoplasmic reticulum (ER) stress, and unfolded protein response (UPR), pathways often interconnected. For their survival in a hostile environment, cancer cells are uniquely reliant on the UPR in a way that normal cells are not. Cancer cells adapt to gain advantage from the UPR to prevent apoptosis, favoring tumor progression and resistance to drug treatments [16]. Components of the heat shock protein response as well as of the UPR, often overexpressed in cancer cells to promote resistance to anticancer therapies, could be targeted to tip the balance towards apoptosis. The response to stress is triggered by oncogenic transcription factors such as heat shock factor 1 (HSF1), the UPR transcription program, NRF2 and hypoxia-inducible factor-1 (HIF-1) that may interact with mutp53 enhancing its GOF. Therefore, mutp53 GOF functional activity may vary according to changes within tumor cells or in the tumor microenvironment [12]. In this review, the interplay between mutp53 and the molecular pathways activated in response to stress is discussed, unveiling how their strict interconnection sustains mutp53 oncogenic potential and synergizes with other oncogenic transcription factors and highlighting how their manipulation could improve the clinical outcome of the anticancer therapies of mutp53-carrying cancers.

## 2. Mutant p53 and Heat Shock Factor 1 (HSF1)/Heat Shock Proteins (HSP) Oncogenic Signaling

**Figure 1:**
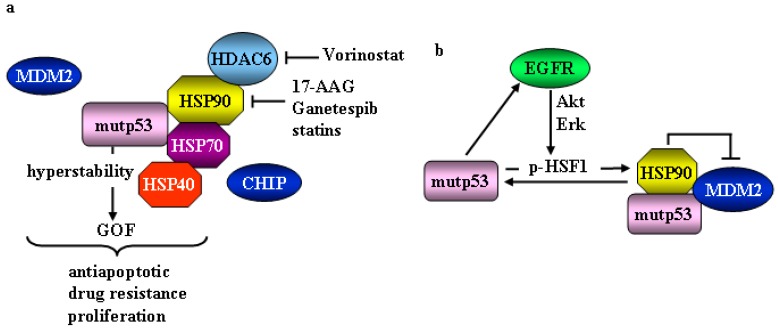
Mutant p53 and heat shock proteins (HSP) machinery. (**a**) Mutp53 protein binds the cellular chaperone HSP90 that protects it from MDM2-dependent degradation. Activation of HSP90 includes deacetylation by HDAC6. Mutp53 stabilization is achieved also by the interaction with chaperone HSP40 and HSP70, that protect mutp53 from CHIP-mediated degradation and cooperate with HSP90 in stabilizing mutp53. The mutp53 hyperstability is necessary for mutp53 oncogenic functions (GOF). Inhibitors of HSP90 and HDAC6 are indicated and allow mutp53 degradation. (**b**) HSP90 transcription is mediated by HSF1. Mutp53 interacts with activated p-Ser326 HSF1 stimulating its transcriptional activity toward HSP90. Then, HSP90 stabilizes mutp53 itself by inhibiting MDM2. Mutp53 drives enhanced recycling of EGFR to the cancer cell surface activating the MAPK and PI3K signaling cascade leading to HSF1 phosphoactivation.

Under normal conditions, wtp53 is targeted by the E3-ubiquitin ligases MDM2, COP1, Pih2, CHIP for ubiquitination and rapid degradation via proteasome. After DNA damage wtp53 undergoes posttranslational modifications-induced stabilization and nuclear translocation for transcriptional activity [17]. Differently from wtp53, mutp53 proteins attain hyperstability because they may acquire a misfolded and partially denatured conformation with high tendency to form micro- and macro-aggregates [18,19] that cannot undergo proteasomal degradation [20,21]. This hyperstability is involved in mutp53 oncogenic function. Mutp53 proteins bind the cellular chaperones heat shock proteins (HSP), such as HSP90, an ATP-dependent molecular chaperone that protects several proteins, including mutp53, from proteolytic degradation [22]. The interaction of mutp53 with HSP90 inhibits MDM2 ubiquitin-protein isopeptidase ligase function by concealing the ARF-binding site on MDM2, resulting in the stabilization of both mutp53 and MDM2 [23]. Moreover, HSP90 inactivates CHIP, severely impairing the degradation of mutp53 [24]. HSP90 is aberrantly activated or upregulated in cancer cells, compared to the normal ones, therefore is considered an important target for anticancer therapy [25]. The inhibition of HSP90 by geldanamycin derivative 17-allylamino-demethoxy geldanamicyn (17-AAG) [26] or by new generation inhibitor ganetespib [27] has been shown to release mutp53 from the complex, enabling efficient p53 degradation [24]. Of note, HSP90 inhibition may sensitize cancer cells to different therapies [28]. HSP90 functional activation includes deacetylation by HDAC6 and its inactivation by HADC inhibitor vorinostat (SAHA) or by HDAC6 knockdown leads to HSP90 hyper-acetylation with loss of HSP90 chaperone activity and release of mutp53 which can undergo degradation [21,29] (Figure 1a). HSP90 transcription is mediated by heat shock factor 1 (HSF1), the master regulator of the heat shock response, basally activated in cancer cells to cope with proteotoxic stress due to aneuploidy, oxidative stress and reactive oxygen species (ROS), hypoxia and acidosis [30,31]. HSF1, other than maintaining cellular homeostasis by stress-mediated induction of HSP, coordinates cellular processes critical for malignancy, such as metastasis, cell cycle control, and inhibition of apoptosis [31,32]. Not surprisingly, HFS1 is upregulated in many types of cancers that hijack HSF1-dependent pathways to promote their own survival and its targeting is a promising strategy to combat cancer [32,33]. Intriguingly, several mutp53 proteins (i.e., R273H, R175H, R280K) induce HSF1 upregulation, and mutp53R280K has been shown to interact with activated p-Ser326 HSF1 stimulating its transcriptional activity toward HSP90 and HSP70, in breast cancer cells [30]. Then, HSP90 stabilizes mutp53 itself in a positive feed-forward loop that reinforces the mutp53/HSF/HSP oncogenic signaling [30]. The HSF1 activation by mutp53 can be stimulated by deregulated MAPK signaling [34]. Thus, mutp53 drives enhanced recycling of EGFR to the cancer cell surface [35] activating the MAPK and PI3K signaling cascade leading to HSF1 phosphoactivation [36] (Figure 1b). These findings may have clinical implications because the chemoresistance induced by mutp53-carrying cancer cells [11] may be, at least in part, counteracted by targeting the mutp53/HSF1 regulatory loop. The proof-of-concept of the nature of mutp53 pro-oncogenic function modifiers comes from in vivo studies in transgenic *Hsf1*-knockout, *Trp53R172H* (equal to R175H in human) mice that do not develop cancer [37]. Inhibition of EGFR, Her2 or of their downstream effectors can interrupt the mutp53-HSF1- Her2/EGFR circuitry in cancers bearing Her2/mutp53 double positive mutation (i.e., breast cancer, pancreatic cancer, and non-small cell lung cancers) [11].

Mutp53 stabilization is also achieved by the interaction with chaperone DNAJA1, an Hsp40 family member that protects mutp53 from CHIP-mediated degradation [38]. Both Hsp40 and CHIP interact with Hsp70 that cooperates with HSP90 in stabilizing mutp53 [39] (Figure 1a). The latter regulatory mechanism can be disrupted by statins, cholesterol-lowering drugs, that, by reducing the level of mevalonate-5-phosphate in the mevalonate pathway (MVP), induce CHIP-mediated degradation of mutp53 [38]. MVP is required for mutp53-Hsp40 interaction, and its upregulation is one of the mutp53 GOF by activating the sterol biosynthesis master transcription factor SREBP [40]. The MVP produces sterols and isoprenoids required for the synthesis of membranes and lipid rafts, signal transduction and protein prenylation that are integral to tumor growth and progression [41]. The interplay between mutp53 and MVP also promotes tumor cells’ aberrant mechano-responsiveness and tumor invasion through activation of YAP/TAZ and geranylgeranylation pyrophosphate (GGPP)-dependent RhoA activation [42,43]. On the contrary, wtp53 downregulates the MVP to induce tumor suppression [44]. Thus, p53 blocks activation of SREBP-2, the master transcriptional regulator of the MVP, by transcriptionally inducing the *ABCA1* cholesterol transporter gene [44]. These findings demonstrate that the oncogenic cooperation of mutp53 with the HSP machinery by HSF1 interaction renders cancer cells more resistant to proteotoxic stress providing a strong survival advantage to cancer cells that acquire chemoresistance and invasion ability [45]. Therefore, pharmacological blockade of mutp53-stabilizing mechanisms by eliciting mutp53 degradation may restrain tumor growth in mutp53-carrying tumors and increase tumor-free survival of mutp53 knock-in mice [21,22,24,45].

## 3. Mutant p53 and the Endoplasmic Reticulum (ER) Stress/Unfolded Protein Response (UPR)

**Figure 2:**
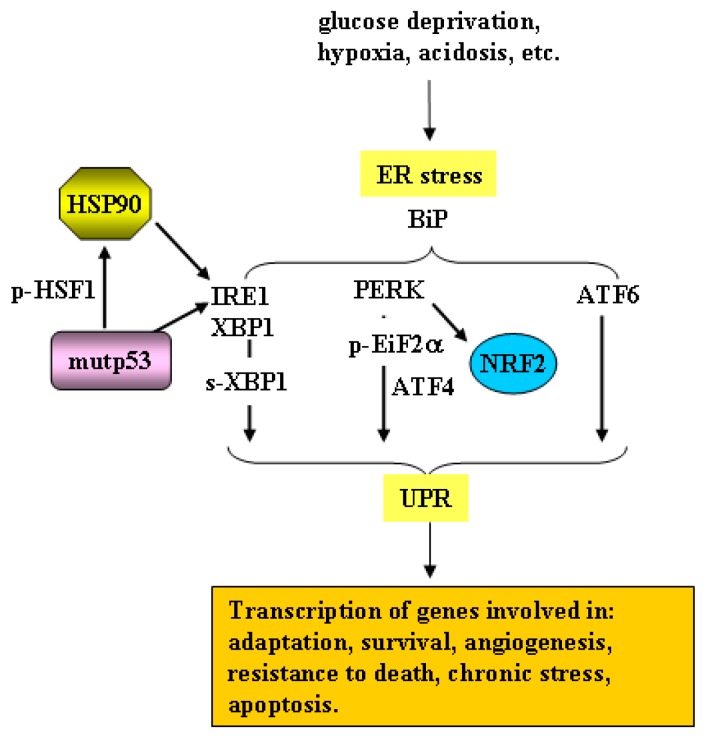
Molecular mechanisms of ER stress unfolded protein response (UPR) pathways. Following internal or external stimuli that induce ER stress, BiP detaches from the three main sensors of UPR, localized at the ER membrane, and active them: inositol-requiring enzyme 1α (IRE1α), PKR-like ER kinase (PERK), and activating transcription factor 6 (ATF6). IRE1α activation induces XBP1(S) transcription factor, PERK phosphorylates and activates NRF2 and eIF2α, ATF6 is translocated to and is processed at the Golgi apparatus to create a highly active transcription factor. All three transcription factors upregulate chaperones and specific targets genes involved in protein folding, antioxidant response, autophagy, and apoptosis, to restore ER homeostasis or to induce cell death pathways. HSP90, which is involved in mutp53 stabilization, may modulate UPR through its association with the cytoplasmic domain of IRE1 and PERK. Mutp53 may activate HSF1 to promote HSP90 induction, highlighting a link between mup53, HSF1/HSP, and UPR.

The endoplasmic reticulum (ER) is the principal intracellular organelle responsible for protein folding, localization and post-translational modifications [46]. Under normal growth condition, the unfolded protein response (UPR) ensures the maintenance of cellular homeostasis thereby controlling the cell-fate decision under ER stress [47]. ER stress is activated by a variety of factors in the intracellular or extracellular compartment, i.e., glucose deprivation, hypoxia, acidosis, inhibition of protein glycosylation, disturbance of intracellular Ca2+ stores, promoting the accumulation of unfolded or misfolded proteins in the ER [48,49]. To cope with ER stress, cells activate the HSP machinery and the UPR to reduce the amount of misfolded proteins through ubiquitin-proteasome-dependent ERAD (ER-associated degeneration) and autophagy systems [50,51]. The UPR transcriptional program that induces adaptation, survival, transformation, angiogenesis, and resistance to cell death [52], is regulated by three main sensors localized at the ER membrane: the inositol-requiring enzyme 1α (IRE1α), PKR-like ER kinase (PERK) and the activating transcription factor 6 (ATF6) [47] (Figure 2). Under basal condition, an ER luminal chaperone protein, GRP78/BiP (glucose-regulated protein 78/binding immunoglobulin protein), binds to these molecules from the ER lumen to suppress their activation [53]. During ER stress, misfolded proteins in ER bind BiP preventing its inhibitory binding to IRE1α, PERK, and ATF-6, thus leading to their activation [54]. IRE1α and PERK undergo oligomerization and trans-autophosphorylation [55] while ATF6 moves from the ER to the Golgi apparatus where undergoes cleavage in the soluble active form that migrates to the nucleus to activate transcription [56]. The IRE1α trans-autophosphorylation induces a conformational change with endoribonuclease activation leading to expression of the transcription factor XBP1(S) by a non-conventional XBP1 mRNA splicing [57]. XBP1(S) increases the expression of ER chaperones and ER mass, stimulates lipid biogenesis, and degrades unfolded proteins to enhance the secretory function of ER to suppress ER stress-mediated cell death [47,58]. The unbalance of the components of the UPR system may contribute to several pathologies including cancer [59]. In this regard, the activation of IRE1/XBP1 pathway is involved in the progression of different tumors including glioblastoma, triple-negative breast cancer and multiple myeloma [60] and may contribute to metastatic progression and chemoresistance [61] Interestingly, depletion or knockdown of p53 has been shown to increase IRE1α/XBPI pathway and BiP expression, in the presence or absence of ER stress, as p53 drives IRE1α protein to degradation through proteasome, while ATF6 and PERK/eIF2a pathways are suppressed [62]. BiP overexpression has been shown to suppress PERK and ATF6 activation, rendering cells resistant to ER stress [56,63], suggesting an aberrant UPR activation under p53 depletion. In line with this evidence, p53 null cells are resistant to ER stress-induced cell death. Therefore, pharmacological inhibition of IRE1α in p53-null cancer cells abolishes such resistance and significantly reduces tumor growth in an in vivo mouse model [62]. Intriguingly, IRE1α is overexpressed in cancer cells lines expressing mutp53, compared to cells expressing wild-type p53, linking mutp53 to the adaptation of tumor cells to ER stress and highlighting an important proof-of-concept for targeting UPR, particularly the IRE1/XBP1 pathway, in tumors harboring mutant p53 [62].

Interestingly, HSP and UPR are inter-connected responses [64]. Indeed HSP90, which is involved in mutp53 stabilization in tumor cells, may modulate the transcriptional arm of UPR through its association with the cytoplasmic domain of IRE1 and PERK [65], as described for other HSP90 client type I transmembrane kinases [66,67,68,69]. HSP90 inhibition with geldanamicyn (that, as said above, promotes mutp53 degradation) induces the ER stress response with reduction of IRE1 protein and increase of BiP expression [65]. As mutp53 may activate HSF1 to promote HSP induction [30], the above findings highlight a link between mup53, HSF1/HSP, and UPR, molecules cooperating in the adaption of cancer cells to proteotoxic stress to allow their survival (Figure 2). More recently, by comparative gene expression profiling of p53-mutated and p53-depleted cancer cells, ectonucleoside triphosphate diphosphohydrolase 5 (ENTPD5) has been identified as a new mutp53 target gene. ENTPD5 promotes the folding of N-glycosylated membrane proteins in the calnexin/calreticulin cycle of the ER, ensuring enhanced expression of membrane receptors. Mutp53 is recruited by Sp1 to the ENTPD5 core promoter to induce its expression. ENTPD5 overexpression is another pro-tumorigenic effect of mutp53, and therefore it might represent another promising target for the treatment of tumors harboring p53 GOF mutations [70]. These findings suggest that a better understanding of the interplay between mutp53, HSF1/HSP, and UPR response may unveil new possible druggable targets to reduce cancer cell adaptation to stresses and thus tumor progression and chemoresistance [16].

## 4. Mutant p53, Nuclear Factor Erythroid 2-Related Factor 2 (NRF2), Hypoxia-Inducible Factor-1 (HIF-1) and Estrogen Receptor α (ERα) Oncogenic Signaling

**Figure 3:**
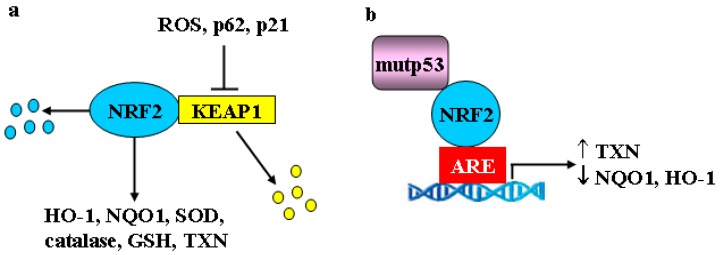
Mutant p53 and the NRF2 signaling. (**a**) NRF2 undergoes protein degradation following interaction with KEAP1 that can be released by oxidative stress and reactive oxygen species (ROS) generation or by p62- and p21-induced noncanonical KEAP1 degradation. (**b**) Mutp53 may interact with NRF2 and bind the antioxidant response elements (ARE) of target genes and differentially modulate NRF2 transcriptional activity, increasing thioredoxin (TXN) and reducing NAD(P)H:quinone oxidoreductase 1 (NQO1) and heme oxygenase 1 (HO-1) expression.

Cancer cells adapt and survival to various forms of oxidative, electrophilic, thermal and inflammatory stress by activating not only the HSF/HSP response and UPR but also the nuclear factor erythroid 2-related factor 2 (NRF2) encoded by the *NFE2L2* gene, considered the major regulator of the antioxidant response [71]. Both HSF1 and NRF2 trigger a transcriptional program for the maintenance of cellular structure, redox, and intermediate metabolism, as they share transcriptional targets such as p62, ATF3, HSP70, and heme-oxygenase 1 (HO-1) [72]. Although transient activation of HSF1 and NRF2 is undoubtedly broadly cytoprotective, their persistent activation may promote tumor progression and chemoresistance. During oxidative and electrophilic stress, canonical NRF2 activation is achieved by release from its negative regulator KEAP1 (Kelch-like ECH-associated protein 1). Then, stabilized NRF2 moves to the nucleus where heterodimerizes with small Maf proteins or ATF4 to bind to anti-oxidant responsive elements (ARE) in the DNA promoter of target genes, including phase I (i.e, aldoketoreductases), phase II and phase III (drug transporters and drug efflux pumps) detoxifying enzymes (Figure 3a). The phase II detoxifying enzymes include catalase, superoxide dismutase (SOD), HO-1, NAD(P)H quinone oxidoreductase 1 (NQO1), and glutathione (GSH), that help to restore cellular redox homeostasis [73]. NRF2 may also repress genes that promote apoptosis [71] and, for that reason, it is considered a “double face” molecule having both tumor suppressive and tumor-promoting effects, the latter being described as the “dark side of NRF2” [74,75,76]. Because of the protective role of NRF2 transcriptional program, controlled activation of this pathway, as well as of the oxidative stress, has been recognized as a means for chemoprevention [77]. Constitutive activation of NRF2, mainly due to inactivating mutations on the *KEAP1* gene or gain-of-function mutations on the *NFE2L2* gene, that occur in several different kinds of tumors (e.g., lung, melanoma, hepatocellular carcinoma), may act as a driver of cancer progression, metastasis, and resistance to therapies [71]. Therefore, targeting NRF2 is considered a helpful strategy to halt tumor growth [78]. Among the NRF2 targets involved in chemotherapeutic resistance, there is catalase, whose overexpression protects cancer cells from apoptosis induced by DNA-damaging agents [79,80] and NQO1, whose inhibition potentiates the cytotoxicity of anticancer treatments [81,82]. The NRF2 pathway can intersect with the autophagy pathway, a catabolic process also activated during various conditions of cellular stress, including nutrient deprivation or DNA damage. Autophagy may help to eliminate unfolded proteins or damaged organelles, promoting cell survival in the face of bioenergetic stress even if, in some instances, it may induce cell death [83]. In particular, the bridging molecule between NRF2 and autophagy is p62 (also called sequestosome 1 -SQSTM1), an autophagy adaptor protein transcribed by both HSF1 and NRF2. P62 is mainly degraded through autophagy and is thus considered a marker of efficient autophagic flux [84,85]. Among several functions, p62 induces KEAP1 degradation through autophagy, thereby triggering noncanonical NRF2 stabilization and activation [86] (Figure 3a). NRF2 activation can thus be a consequence of autophagy reduction and may, as said above, promote tumor progression and chemoresistance. Another noncanonical activation of NRF2 can be mediated by the p53 target p21^Cip1/WAF1^ that directly interacts with NRF2 interrupting the KEAP1/NRF2 complex, leading to NRF2 stabilization and upregulation of the NRF2 signaling pathway, under both basal and induced stressful conditions [87] (Figure 3a). In addition, the ER stress-induced PERK activation may phosphorylate and activate NRF2 [88] linking ER stress/UPR to the oxidative stress signaling, both involved in the expression of pro-survival genes [89] (Figure 2).

Recent findings showed that mutp53 may interact with NRF2 and differentially modulate NRF2 transcriptional activity. This selected activation of the NRF2 transcriptional pathway allows cancer cells to manage high levels of intracellular ROS, promoting a pro-survival antioxidant response [90,91]. Thus, the mutp53-NRF2 interaction upregulates thioredoxin (TXN) that is associated with poor prognosis in breast cancer, as its silencing decreases survival and migration of breast cancer cells. Conversely, the mutp53-NRF2 interaction prevents NRF2 binding to other targets, such as NQO1 and HO-1, reported to induce cytotoxic effects in cancer cells, as demonstrated by their silencing that increases breast cancer cell survival and migration (Figure 3b) [90,91]. The proof-of-principle of the pro-oncogenic function of mutp53-NRF2 interaction comes from in vivo studies of carcinogenesis and invasion in a mouse model of pancreas-specific mutant K-ras and p53 double mutant, where deletion of NRF2 decreases the formation of pancreatic carcinogenesis and invasion [92]. In accordance, a combination therapeutic strategy using the specific TXN inhibitor, Auranofin, and APR-246 drug targeting mutp53, synergistically reduces breast cancer cell growth [91]. The oncogenic interaction between mutp53 and NRF2 has been also shown in an interesting study from the same authors demonstrating that five different GOF mutants, in the context of their respective triple negative breast cancer (TNBC) cell lines, cooperate with NRF2 to induce transcription of 20S/26S proteasome/immunoproteasome gene, as a new GOF program of mutp53 [93]. This unusual overexpressed signature enhances the degradation of tumor suppressor proteins and confers resistance to proteasome inhibitor therapy. Therefore, combined inhibition of mutp53 with APR-246/PRIMA1-Met and of the proteasome with carfilzomib may effectively overcome this resistance mechanism [93]. 

To make the link between NRF2 and mutp53 even more complex, it has been recently shown that mutp53 may cooperate with hypoxia-inducible factor 1 (HIF-1). HIF-1 is a heterodimeric transcription factor that consists of the HIF-1β subunit, constitutively expressed in cells, and the HIF-1α subunit whose stability is stimulated by low intracellular oxygen or genetic alterations [94]. HIF-1 is considered the main effector of the cellular response to hypoxia inducing the transcription of target genes involved in many aspects of cancer progression, including angiogenesis, metabolic adaptation, chemoresistance, apoptosis evasion, invasion and metastasis, therefore, its targeting is considered a useful anticancer strategy [95,96]. Mutp53 physically interacts with HIF-1α subunit and shuttles it to the DNA genomic elements impinging on HIF-1 transcriptional activity, thus transcriptionally regulating a gene expression signature involved in tumor progression of non-small cell lung cancer, in vitro and in vivo [97]. Intriguingly, both HIF-1 and NRF2 are activated in response to hypoxia, likely due to the formation of hypoxia-induced ROS, and both cooperate to promote metastasis and play complementary roles in chemoresistance [98]. Knockdown of NRF2 or its pharmacologic inhibition by drugs such as brusatol [99,100,101] downregulates HIF-1α promoting its proteasomal degradation, highlighting the therapeutic potential of NRF2 targeting to counteract the hypoxia-induced chemoresistance. However, the most successful strategy to overcome chemoresistance seems to be simultaneous targeting of both pathways, i.e., by triptolide, a molecule derived from Chinese herbal extract [102]. 

In this context, it is worth to discuss that one of the main tumor promoter roles of NRF2 is its ability to induce cancer cell proliferation and aggressiveness by affecting cancer cell metabolism. Thus, NRF2 redirects glucose and glutamine into anabolic pathways, in particular under the sustained activation of PI3K-Akt signaling [103]. In addition, NRF2 controls the expression of the key serine/glycine biosynthesis enzyme genes PHGDH, PSAT1 and SHMT2 via ATF4 to support glutathione and nucleotide production. This signature confers poor prognosis in human non-small cell lung cancer cells [104]. Metabolic reprogramming is a hallmark of cancer cells and plays a role in tumor progression [105]. In this regard, mutp53 activates glycolysis in cancer cells to promote cancer cell growth, another GOF for mutp53 [106], suggesting that unveiling the link between mutp53 and NRF2 oncogenic signaling may reveal additional druggable targets to manipulate the metabolic reprogramming and arrest tumor growth.

To further link these pathways together, a reciprocal interplay has been shown between HIF-1 and Estrogen receptor-α (ERα). Thus, HIF-1α is a direct transcriptional target of ERα [107]. A majority of breast cancers are driven by estrogen via ERα, a nuclear transcription factor that is critical for mammary epithelial cell division and breast cancer progression thus, inhibiting Estrogen receptor action by reducing the estrogen levels may be beneficial for early-stage patients and those with advanced disease [108]. These two pathways may act in cooperation to promote breast cancer progression, as assessed by clinical studies showing that HIF-1α and hypoxia gene signature were correlated with poorer survival in response to hormone therapy [107]. These results complement findings showing that, in response to UPR or ER stress, XBP1 might interact with HIF-1α to confer antiestrogen resistance in triple negative breast cancer [109]. Intriguingly, some studies showed that estrogen receptors are able to act in *cis* with p53 at canonical, as well as noncanonical p53 target sequences to enhance transactivation and this synergy applies to cancer-associated p53 mutants, greatly expanding the transcriptional master network regulated by p53 in terms of genes affected and levels of expression [110]. These findings further link mutp53 with HIF-1 through ERα. Intriguingly, knockdown of ERα has been shown to induce autophagy and inhibit antiestrogen-mediated UPR activation, promoting ROS-induced breast cancer cell death [111]. It would be interesting to evaluate, whether, in this context mutp53 undergoes autophagy-mediated degradation, to link the pathways together for therapeutic purpose. Thus, these findings suggest that the relationship between mutp53, ERα/HIF-1 under conditions of chronic stress, may be a druggable pathway to increase the efficacy of endocrine therapy in breast cancers.

Altogether, these findings underline the strict relationship between mutp53, HSF1, NRF2 and HIF-1/ERα signaling pathways that reinforce each other, increasing their oncogenic potential. Given the interplay among them, it is conceivable that blocking one pathway may influence the other pathways and that synchronously blocking them may have greater success in anticancer therapy.

## 5. Mutant p53 and Autophagy: Linking the Pathways Together

**Figure 4:**
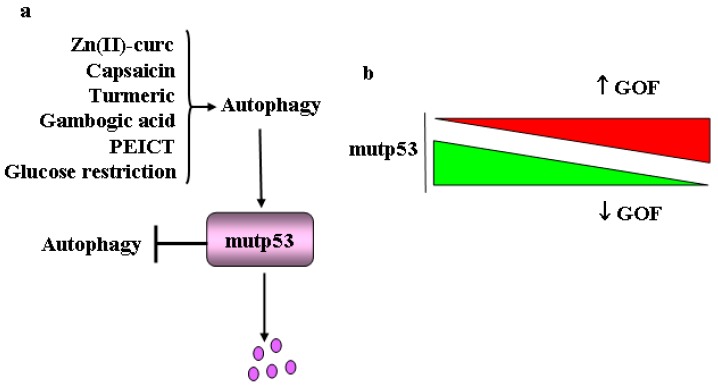
Interplay between mutp53 and autophagy and the effect of mutp53 stability/degradation. (**a**) Mutual interplay between autophagy and mutp53: autophagy degrades mutp53 and mutp53 inhibits autophagy counteracting its own elimination. (**b**) Schematic representation of mutp53 GOF depending on mutp53 expression: the reduction of mutp53 expression (green triangle) reduces GOF while increased mutp53 expression (red triangle) increases GOF.

In the last years, direct mup53 protein-targeting drug strategies have been developed at various preclinical levels for mutp53 degradation and/or wtp53 reactivation. Pleiomorphic degradation is the most advanced strategy that shows impressive survival effects in mutp53 knock-in mouse models [11]. We have shown that mutp53 degradation can occur through autophagy activation. This effect was obtained by natural compounds, such as Zn(II)-curcumin and capsaicin, that, by acting on protein folding, reactivate wtp53 that induces its target gene *DRAM* (damage-regulated autophagy modulator) promoting autophagy-induced degradation of mutp53 (R175H and R273H mutants) proteins and restoring cancer chemosensitivity [112,113,114,115,116]. Intriguingly, these natural molecules may instigate ER stress and reduce HSP90 expression, all effects favoring mutp53 degradation [117]. Other natural compounds have been reported to induce mutp53H273 protein degradation through autophagy, including the dietary spice turmeric, and its bioactive component curcumin [118]. Gambogic acid, a potent apoptotic molecule, may stimulate the degradation of mutp53 through autophagy and consequently increase the response to chemotherapies [119], although it may also induce protein degradation through proteasome ubiquitination by CHIP [120]. Similarly, cruciferous-vegetable-derived phenethyl isothiocyanate (PEICT) renders p53H175 sensitive to degradation by both proteasome and autophagy [121]. We found that apigenin activates wtp53 [122] and, intriguingly, it also induces mutp53 degradation through autophagy, involving ROS generation and NRF2 activation [117]. Another important strategy to induce mutp53 degradation via autophagy, both in vitro and in vivo, is glucose restriction (Figure 4a) [123] and long-term nutrient deprivation that has been reported to induce mutp53 degradation by selective chaperone-mediated autophagy (CMA) [124]. Finally, inhibition of MKK3, a dual specificity protein kinase that belongs to the MAP kinase kinase family, reduces mutp53 protein levels through ER stress-induced autophagy [125].

Interestingly, a mutual interplay between autophagy and mutp53 occurs, as on one hand autophagy degrades mutp53 rendering the cancer cells free from this oncogene and, on the other hand, mutp53 inhibits autophagy counteracting its own elimination, and promoting chemoresistance [126] (Figure 4a). Thus, the repression of autophagy is functional to the pro-survival and anti-apoptotic GOF of p53 mutants (Figure 4b) [127]. The inhibition of autophagy by mutp53 is achieved by several mechanisms including inhibition of beclin 1 and ATG12 or reduction of AMPK and activation of mammalian target of rapamycin (mTOR)/PKM2 signaling [127,128]. mTOR, a highly conserved serine/threonine kinase, a downstream effector of PI3K/Akt or Ras/MAPK pathways, is a key regulator of the autophagic pathway [129,130]. It comprises two different complexes, namely mTOR complex 1 (mTORC1) and mTOR complex 2 (mTOR2), the former being the main regulator of autophagy that can be directly targeted by rapamycin or its analog everolimus. mTORC1 inhibition, which can also occur under nutrient starvation, stimulates autophagy whereas its activation, i.e., in nutrient-rich conditions, reduces this process [131,132,133]. To date, pharmacological induction of autophagy, i.e., through mTOR inhibition or AMPK activation, has been shown to have some potential in cancer prevention and therapy [134]. Interestingly, the mutp53-induced stimulation of the mTOR pathway, that inhibits autophagy, has been shown to sensitize cancer cells to mTOR inhibitor everolimus [127]. Intriguingly, the same authors showed that mutp53 may modify autophagy gene transcription by its interaction with p50 NF-kB subunit, opening new avenues in the developing of new drugs that inhibit this oncogenic mechanism and restore autophagy [127]. Of note, mutp53 promotes glycolysis by enhancing mTORC1-mediated phosphorylation of glycolytic enzyme PKM2, sustaining tumor progression [128]. 

Given the interplay between mutp53 and NRF2/HIF-1, between ERα and HIF-1, between mutp53 and autophagy, and between NRF2/HIF and autophagy, it is conceivable to hypothesize that mutp53 stabilization could promote NRF2 and ERα/HIF-1 activation also by reducing autophagy. In the case of NRF2, because it is stabilized by p62 that accumulates following autophagy inhibition by mutp53 and, in the case of HIF-1, because its lysosomal degradation may be reduced by mutp53. On the other hand, the oncogenic potential of these partners in crime may be dismantled by induction of autophagy to degrade mutp53 and disrupt the connected complexes like falling dominoes. Of note, differently from the other pro-survival pathways that are activated by mutp53, autophagy seems to be negatively regulated by this oncogenic protein. This may represent a self-protective mechanism, as autophagy contributes to mutp53 degradation. For this effect, for the autophagy capacity to degrade other oncogenic proteins other than mutp53, such as HIF-1 and c-myc [135] and for its role in promoting the immunogenic cell death [134,136], autophagy might turn out to be a process that possibly fights cancer, instead of promoting it, as it has been for a long time considered [137]. 

## 6. Conclusions

Many different types of cancer show a high incidence of *TP53* mutations and some of them may acquire oncogenic functions that underline the aggressive phenotype of breast cancer, pancreatic cancer, and non-small cell lung cancer, therefore providing numerous exciting therapeutic possibilities [138]. Given its pivotal role in oncogenesis, mutp53 protein-targeting drug strategies, aimed at inducing its degradation and/or restoring wtp53 function, are currently being developed at various preclinical levels [11,139]. However, mutp53 may interact with transcription factors involved in the cell response to stress, including HSF1, NRF2, and HIF-1, that reinforce each other, increasing their oncogenic potential, making the targeted therapies even more challenging. Therefore, it is conceivable that blocking one pathway may influence the other pathways and that synchronously blocking them may have more profound consequences than anticipated.

## Abbreviations

ARE: anti-oxidant responsive elements; ENTPD5, ectonucleoside triphosphate diphosphohydrolase 5; ER, endopasmic reticulum; ERα, estrogen receptor-α; GOF, gain-of-function; GRP78/BiP, glucose-regulated protein 78/binding immunoglobulin protein; GSH, glutathione; HIF-1; HO-1, heme-oxygenase 1; HSF1, heat shock factor 1; HSP, heat shock protein; IRE1α, inositol-requiring enzyme 1α; mTOR, mammalian target of rapamycin; mTORC1/2, mTOR complex 1/2; mutant p53, mutp53; MVP, mevalonate pathway; NQO1, NAD(P)H:quinone oxidoreductase 1; NRF2, nuclear factor erythroid 2-related factor 2; PERK, PKR-like ER kinase; ROS, reactive oxygen species; SOD, superoxide dismutase; TXN, thioredoxin; UPR, unfolded protein response; wtp53, wild-type p53.
